# New Aspects on Tick-borne Encephalitis in Children

**DOI:** 10.1097/INF.0000000000005074

**Published:** 2025-12-08

**Authors:** Kacper Toczylowski, Dawid Lewandowski, Artur Sulik

**Affiliations:** From the * Department of Pediatric Infectious Diseases, Medical University of Bialystok, Bialystok, Poland.

**Keywords:** tick-borne encephalitis, flavivirus, cognitive dysfunction, meningitis, encephalitis

Tick-borne encephalitis (TBE) is an emerging viral neuroinvasive disease transmitted primarily through tick bites, occurring across Europe and Asia. Recent epidemiologic data reveal a significant shift in our understanding of TBE in children. While the traditional view held that children experience milder disease with better outcomes than adults, they can still develop serious neurological sequelae or long-term cognitive deficits following infection. Particularly noteworthy is the recent recognition of subtle neurocognitive impairments that may go undetected during standard neurological evaluations but can substantially affect a child’s academic performance, quality of life and future potential. This review summarizes the current knowledge on TBE and outlines practical strategies for managing the disease in children.

## Classification

Tick-borne encephalitis virus (TBEV) is the widely recognized name for the virus now classified as the species *Orthoflavivirus encephalitidis*, following the updated taxonomy established by the International Committee on Taxonomy of Viruses.^1^ The virus belongs to the *Flaviviridae* family. Three genetic subtypes of TBEV are classically recognized: Far-Eastern (TBEV-FE), Siberian (TBEV-Sib) and European (TBEV-Eur). Recent studies have identified new Baikal and Himalayan subtypes and genetic variations within existing subtypes. The reported data suggest variability in outcomes across different virus subtypes. However, direct comparisons are limited due to inconsistent reporting and unspecified subtypes in some studies. Clinical presentations suggest that the Far-Eastern subtype typically manifests with severe central nervous system involvement and higher mortality rates. The Siberian subtype shows varied clinical presentations, including reports of chronic TBE forms not observed with other subtypes. Data specifically in children are scarce, but available evidence indicates children fare better than adults in all subtypes (with extremely rare fatalities in children with the European subtype), yet the pattern of subtype severity reflects that in adults.^2^

## Epidemiology and Surveillance

TBE reported incidence could be incomplete for many reasons, just to mention some: most cases are asymptomatic or mild, serology testing could be influenced by previous vaccination, the real risk of exposure is difficult to assess, given little is known about reservoir animals across the continent. In children, cases are even more frequently overlooked compared to adults due to milder or atypical symptoms and diagnostic challenges. In Europe, traditionally, TBE is reported extensively from countries in the central and eastern part of the continent, but it appears that currently, TBE is expanding in every direction. Incident rates increase in northern Scandinavia, indigenous cases are reported from the United Kingdom,^3^ the Netherlands^4^ and Italy.^5^ Recently, TBEV has even been detected in ticks collected in Tunisia, North Africa.^6^ TBE has long been recognized in parts of Far Eastern Asia, particularly in eastern Russia, northeastern China and northern Japan (Hokkaido). New geographic distribution of the disease is reported from this part of the world as well. It could be partly caused by increased surveillance and awareness, although environmental changes could also be a contributing factor. Climate change has emerged as a critical factor influencing the expansion of TBE endemic areas and increasing case numbers. Warming temperatures accelerate tick development cycles, increase its reproduction rates and extend the tick activity season.^7^

## Transmission

TBE is primarily transmitted to humans through the bite of infected ticks. Children who spend more time playing outdoors in grassy or wooded areas increase their chances of encountering infected *Ixodes* ticks, whereas adults may be at higher risk due to occupational exposure.

A small percentage of cases may be caused by consuming of infected unpasteurized dairy products, mainly of goat or sheep origin. These cases could occur in family outbreaks following the purchase of milk or cheese from animal breeders. Children in the food-borne transmissions had increased risk of TBE infection over adults.^8^ This is mainly because children consume higher quantities of milk relative to their body weight. Alimentary TBE may diverge from that acquired via tick bites, with some studies suggesting a milder course of the disease.^9^

The time from a tick bite to the onset of illness is usually 7–14 days, but can range from 2 days to 4 weeks. For infections acquired through the alimentary route (consuming infected dairy products), the incubation period may be shorter. There are documented extremely rare infections transmitted from mother to child during pregnancy or via substances of human origin. That includes 2 cases of transfusion-transmitted TBEV infection^10^ and 3 patients who received solid organ transplants from a single donor.^11^ A case of probable transmission through breast milk has also been described.^12^

## COURSE OF DISEASE IN CHILDREN

### Initial (Nonspecific) Phase

The disease shows biphasic characteristics, but studies show this pattern occurs less often in children than adults, with variable rates from 58% to 100%. In the initial phase, many infections remain asymptomatic, subclinical, or present with mild, nonspecific or flu-like symptoms, which are often misdiagnosed. In infants and neonates, the disease may progress without an asymptomatic period, with early symptoms including fever, irritability, poor feeding, vomiting and tonic-clonic seizures.^13^ Laboratory findings in the first phase frequently reveal thrombocytopenia, leukopenia, pancytopenia and elevated aminotransferases. At this stage, cerebrospinal fluid (CSF) parameters are usually normal.^14^

### Second Phase

During this second, neurologic phase, 5%–30% of children experience fever, fatigue, headaches, along with meningeal symptoms that may be accompanied by focal neurological deficits and altered consciousness.^15^

In case of suspected TBE in children, a lumbar puncture is recommended. CSF analysis in the neurologic phase reveals pleocytosis (>5 cells/μL), with leukocyte counts ranging from several dozen to several hundred. Protein levels in CSF are moderately elevated, while glucose concentrations remain normal.^15^

A hallmark of TBE is the prolonged persistence of central nervous system (CNS) inflammation. Even after the resolution of acute symptoms, follow-up CSF analysis often shows residual abnormalities: pleocytosis generally normalizes within a few weeks, while elevated protein levels may persist for several months after disease onset.^16^ Importantly, follow-up lumbar puncture in TBE is not routine and should be guided by clinical need, like persistent or worsening neurological symptoms (eg, unresolved meningitis/encephalitis, new focal deficits) to rule out alternative diagnoses.

## MAKING THE DIAGNOSIS

Historically, the TBE diagnosis primarily relied on clinical evaluation, CSF analysis, imaging techniques and epidemiological context, supported by varying laboratory analyses including the complement-fixation reaction, the hemagglutination inhibition test, or the virus neutralization test.^13^ More reliable diagnostic methods with specific antibodies against TBEV by enzyme immunoassay were introduced in the 1980s.^13^ The European Centre for Disease Prevention and Control established standardized case definitions in 2011, requiring both clinical signs of central nervous system involvement and laboratory confirmation—typically through detection of TBE-specific immunoglobulin M (IgM) and IgG antibodies in serum or CSF, or identification of viral RNA via RT-PCR.^17^

However, direct methods have limited utility in diagnosing TBE. Polymerase chain reaction (PCR) is applicable almost exclusively in the early, febrile stage of infection, particularly during the viremic phase when the virus is present in the blood. Its diagnostic utility significantly decreases once the infection progresses to the CNS phase, when most patients undergo diagnostic work-up. At this stage, viral RNA is rarely detectable in blood or CSF, limiting the role of PCR in confirming TBE-associated neurological disease. Thus, laboratory diagnosis primarily relies on serological testing, with IgM and IgG antibody assays available.

Flaviviridae viruses are widespread globally, leading to serological cross-reactions within members of the family, which can significantly complicate the diagnosis of TBE in certain regions. Additionally, prior Yellow fever of Japanese encephalitis vaccinations can induce cross-reactive antibodies that may be misinterpreted as immunity to TBE.

In vaccinated patients, the most accurate diagnostic criteria for TBE rely on laboratory tests that can distinguish infection-induced antibodies from those produced by vaccination. The nonstructural protein 1-specific IgG enzyme-linked immunosorbent assay (ELISA) is particularly valuable, as infection with the TBE virus induces antibodies to both the whole virus and the nonstructural protein 1, whereas vaccination elicits antibodies only to the whole virus.^18^ However, the availability of this test is limited, as it is not widely offered in routine commercial diagnostics and is mainly accessible in specialized or research laboratories. Thus, breakthrough TBE cases are typically diagnosed via TBEV-specific IgM ELISAs or a 4-fold rise in specific IgG titers.^19^ Additional CSF markers, such as intrathecal antibody synthesis, a CSF-serum IgG index, and granulocyte percentages above 20%, assist in distinguishing TBE from other CNS infections.^20^

### Hospitalization and Outcomes

Hospitalization duration varies from a few days to several weeks, with an average stay being significantly shorter in children than in adults (Table 1).

**TABLE 1.** Comparison of Tick-borne Encephalitis Disease Severity and Vaccine Effectiveness in Children and Adults

Feature	Children	Adults
Acute disease severity	Generally milder; meningitis most common (up to 80%); meningoencephalitis less common (13%–41.4%); severe symptoms (paralysis, seizures) rare (<2%); ICU admission 0%–22%	More severe acute disease; higher rates of encephalitis and meningoencephalomyelitis; ICU admission more frequent; longer hospital stays
Mortality	Extremely rare fatalities (especially with the European subtype)	European subtype: 0.5%–2% mortality; higher with Far-Eastern subtype
Long-term sequelae	Up to 70% with cognitive/neurodevelopmental deficits (eg, memory, concentration, executive function), persistent fatigue and headaches >1 year; subtle deficits may go undetected	Generally higher rates and severity of sequelae than in children
Vaccine effectiveness (VE)	90.8% (≥3 doses, children and adolescents, Switzerland 2005–2022); 66%–91% VE against neurological complications; breakthrough cases rare (0.043–0.057 per 100,000/year in Austria)	95%–99% (adults, various studies); breakthrough cases rare (0.009–0.039 per 100,000/year in Austria); similar or slightly higher VE than in children
Booster recommendations	First after 3 years, then every 5 years after primary series (per product label); some evidence supports 10-year intervals	First after 3 years, then every 5 years after primary series (per product label); some evidence supports 10-year intervals
Justification for vaccination	Disease can cause significant long-term cognitive and neurologic sequelae despite a milder acute course; vaccination reduces severe disease risk 8–10 fold	High risk of severe acute disease and long-term sequelae; vaccination highly effective in preventing severe outcomes

Severe complications of TBE, such as limb and cranial nerve paralysis, or abnormal EEG findings, are rare in children. However, children may experience a broad range of long-term, more subtle complications, often overlooked during standard neurological evaluations. More than half of children report persistent fatigue and headaches lasting over a year. Cognitive and neurodevelopmental impairments affect a large proportion of cases for years after the infection. These problems include deficits in executive functions, such as short-term memory, motor coordination, concentration, and behavioral regulation, which can negatively impact school performance and overall development. Notably, the likelihood of these complications does not correlate with the severity of the acute infection. To objectively evaluate the long-term impact of TBE, standardized questionnaires, such as Wechsler Intelligence Scales, paired with neuropsychologic assessments, are recommended.^21^

### Treatment

Currently, there is no specific antiviral therapy for TBE. Treatment remains symptomatic in both children and adults. Clinical experience suggests that dexamethasone may be beneficial in severe pediatric TBE cases by reducing inflammation in the CNS. In vitro studies indicate potential treatment options for the future, including for instance, efavirenz and tipranavir, dasabuvir, or plant-based flavonoids.^22,23^

### Prevention

Healthcare providers can prevent TBE by advising vaccination, personal protective measures, and environmental control.^24^ Vaccination in children is strongly justified by disease severity data and risks of long-term complications. Current evidence demonstrated 90.8% vaccine effectiveness (VE) for complete pediatric vaccination (≥3 doses).^25^ Despite dangers posed by TBE, the uptake of the vaccine remains relatively low in Europe. In a recent study conducted across 20 European countries, the TBE vaccine uptake was on average 22% in endemic and 5% in nonendemic countries. Physician recommendations were the strongest driver of TBE vaccination in both endemic and nonendemic regions, followed by personal risk perception and fear of TBE.^26^

Importantly, vaccines are not entirely effective, and breakthrough infections have been documented both in adults and children. In a highly vaccinated Austrian population, the incidence of breakthrough TBE cases in the years 2000–2006 in children under 15 was estimated to be around 0.043–0.057 per 100,000, which was slightly higher compared to 16–49 years age group (0.009–0.039 per 100,000).^27^ Therefore, it is important to suspect and diagnose TBE in both vaccinated and unvaccinated children with suggestive symptoms.

It is essential to combine vaccination with personal protection measures to prevent TBE infections among children.^28^ Major US health authorities such as the Centers for Disease Control and Prevention and Environmental Protection Agency recommend using insect repellents containing N,N-Diethyl-meta-toluamide, picaridin (also known as icaridin) or IR3535 for children of all ages, all of which are considered safe and effective when used according to label instructions. In addition to repellents, children should wear long sleeves and pants in tick-prone areas. Following outdoor activities, thorough body tick checks are important, as early tick removal reduces the likelihood of transmission of bacterial pathogens. However, the TBE virus can be transmitted almost immediately after the tick attaches, as the virus is present in the tick’s saliva and is injected into the host right at the start of feeding.^29^

Environmental control strategies, like landscaping, mowing grass, and removing leaves, reduce suitable habitat. Lining yards with a perimeter of mulch or wood chips creates barriers between tick habitat and areas used by humans, and fencing discourages wild animals, which may carry ticks from browsing in backyards. All this may reduce tick exposure, and thus, TBE.^30^

### Areas for Future Research

Research on pediatric TBE must address several unresolved factors. Children living in endemic regions are likely exposed to TBE, but the true incidence remains unknown due to mild or atypical presentations that are often undiagnosed. Long-term complications can arise regardless of the initial severity of infection, but the underlying mechanisms behind persistent neurological and cognitive sequelae in children remain poorly understood. The extent of vaccine breakthrough infections in immunized children is still debated, with limited data on the causes of vaccination failure.
